# Effect of Urban fringes green space fragmentation on ecosystem service value

**DOI:** 10.1371/journal.pone.0263452

**Published:** 2022-02-10

**Authors:** Yingying Guan, Xueming Li, Songbo Li, He Sun, He Liu

**Affiliations:** 1 Human Settlements Research Center, Liaoning Normal University, Dalian, China; 2 Liaoning Key Laboratory of Physical Geography and Geomatics, Liaoning Normal University, Dalian, China; Gebze Teknik Universitesi, TURKEY

## Abstract

In this study, an urban fringe green space classification system was established to explore the spatiotemporal variation of green space landscape and ecosystem service value (ESV) based on multi-source land-use data of Ganjingzi district from 2000 to 2018. (1) Results show that the total green space area declined from 359.57 to 213.46 km^2^ over the study period. Green space large plaque index (LPI) and class area both gradually declined, whereas the number of plaques (NP) and plaque density (PD) gradually increased, indicating green space landscape fragmentation. (2) Additionally, the value of green space ecosystem services reduced from 397.42 to 124.93 million yuan. The dynamic degree of ESV change in green space increased or decreased moderately, always being < 0 and showing a decreasing trend of ESV. From a spatial variation perspective, dynamic degrees of ESV variation in the western and northern regions with relatively intensive green space were higher than those in the east. Regarding ESV of various green space types, forest land had the highest functional values of ecological regulation and support, whereas arable land provided the highest functional values of production supply. (3) The ecological service function value of green space system is negatively correlated with PD, NP, edge density, landscape shape index, and Shannon’s diversity index, and positively correlated with aggregation index, contagion metrics, and LPI. The correlation coefficient between the climate regulation function of forest and the change of number of plaques is -0.874. The correlation coefficient of the recreation and culture of the wetland to the plaque density change is no less than -0.214.

## Introduction

The urban ecosystem directly or indirectly provides humans with service functions, production, and living materials. The evolution of the urban ecosystem is closely related to human survival and development [1, 2]. In recent years, urban population growth, land use expansion, and human social and economic activities have threatened the urban ecosystem [3–5], reduced the value of ecosystem services, and affected the sustainable development of human society [6, 7]. Ecosystem service value (ESV) evaluation is of great significance to the rational planning of urban construction and the improvement and restoration of urban ecological environment. The issue of urban ecosystem service value evaluation has attracted increasing attention from the academic community [8, 9].

As an important part of urban land use structure, urban green space has a very important ecological service value [10–13]. The ecological service value of urban green space refers to the economic value generated by the ecological service function of the green space [14, 15]. The ecological service functions of the green space system includes the adjustment function of purifying soil water bodies, maintaining carbon and oxygen balance, adjusting local climate, and alleviating urban "heat island effect" [16, 17]; maintain the functions of beautifying the urban landscape, such as biodiversity [18] and the richness of the landscape [19]; meet the daily leisure and entertainment, science education, cultural exchanges, and other functions of recreation and cultural creation of the urban population [20], and the production function [21]. Conducting research on the evaluation of ecological service value of urban green space system, is conducive to people deeply understanding the role of urban green space vegetation, to select and configure green space green plants more scientifically and rationally, promote urban green space system planning, and provide scientific basis for urban ecological construction [18, 22, 23]. Scholars take the urban green space system as the research object, and classify the composition and structure of the urban green space system [17], service functions [24], landscape pattern evolution [25], ecological effects [26], green space ecological service value evaluation methods [27, 28], and other levels are the research perspective [29], which summarizes the relevant research on the evaluation of urban green space system ecological services. The importance of the evaluation of urban green space ecological service value to urban ecological construction and the improvement of urban human settlements was explained [30, 31].

Changes in the ecological service value of urban green space systems are closely related to changes in urban land use structure, and the increase in construction land and the decrease in green space [32]. Urban green space ecological service value evaluation studies mostly take urban land use change research and green space landscape factor change research [33, 34] as research perspectives, utilizing land use change models [35, 36]. The landscape model index [37] uses the comprehensive valuation and trade-off model (InVEST) to evaluate the ecological service value of the urban green space system. Through Erdas, ArcGIS, Fragstats, and other software, the impact of urban green space landscape structure changes on the value of urban green space ecological services are analyzed based on satellite image data, observation data, etc. [25], and qualitatively classify and quantitatively evaluate urban green space ecological service functions. Through land use land cover change models (LUCC), service provision element models (SPEs), etc., the future evolution of urban ecological service supply value is predicted, and theoretical support for urban infrastructure construction and urban future planning is provided [38].

The urban fringe area has gradually developed as a bearing space for urban construction, urban population gathering, and gradually expanding urban space. As the area between the two systems of "city" and "township," the urban fringe has the most sensitive ecological environment, the largest evolution of land structure, and the fastest urbanization development [39]. As urban land invaded the urban fringe area, the land use structure of the urban fringe area changed, urban construction land increased, area of urban green space gradually decreased showing the characteristics of gradual fragmentation, and the ecological environment of the urban fringe area also changed accordingly [40, 41]. Analyzing the importance of green space landscape in urban fringe areas for the evaluation of ecological service value is helpful to provide a theoretical basis for the spatial planning and construction of urban fringe areas.

To sum up, most of the relevant research on the ecological services of urban green space systems is based on the national and regional scales [42]. As the extension area in the process of urban development, Ganjingzi District is a huge change in the urban functional positioning, and the urban green space system is gradually broken, and the impact of urban ecological service value is worthwhile. Pay attention to the analysis of quantitative analysis, facilitates the development of more conducive to urban sustainable development. The lack of urban fringe areas as a research object, an analysis of the evolution of urban green space landscape index, has a long-term value for urban green space ecological services. Research change over time period. This study uses the fastest urbanization development of Dalian city, the coastal city of Dalian, as the research period from 2000 to 2018. Based on the land use data of Ganjingzi district and Dalian city, combined with remote sensing images, the urban green space is classified according to the national land use classification standard, using Erdas, ArcGIS, Fragstats4.3, and other software to calculate the green landscape index (plaque density, number of plaques, edge density, landscape shape index, Shannon’s diversity index, aggregation index, contagion metrics, and large plaque index) and analyze the changes in the green landscape structure of Ganjingzi district from 2000 to 2018; with a view to scientifically and rationally use the urban green space system, give play to the service value of the green space system, achieve sustainable urban development, and provide new suggestions and perspectives.

## Research data and methodology

### Study area

**Fig 1:**
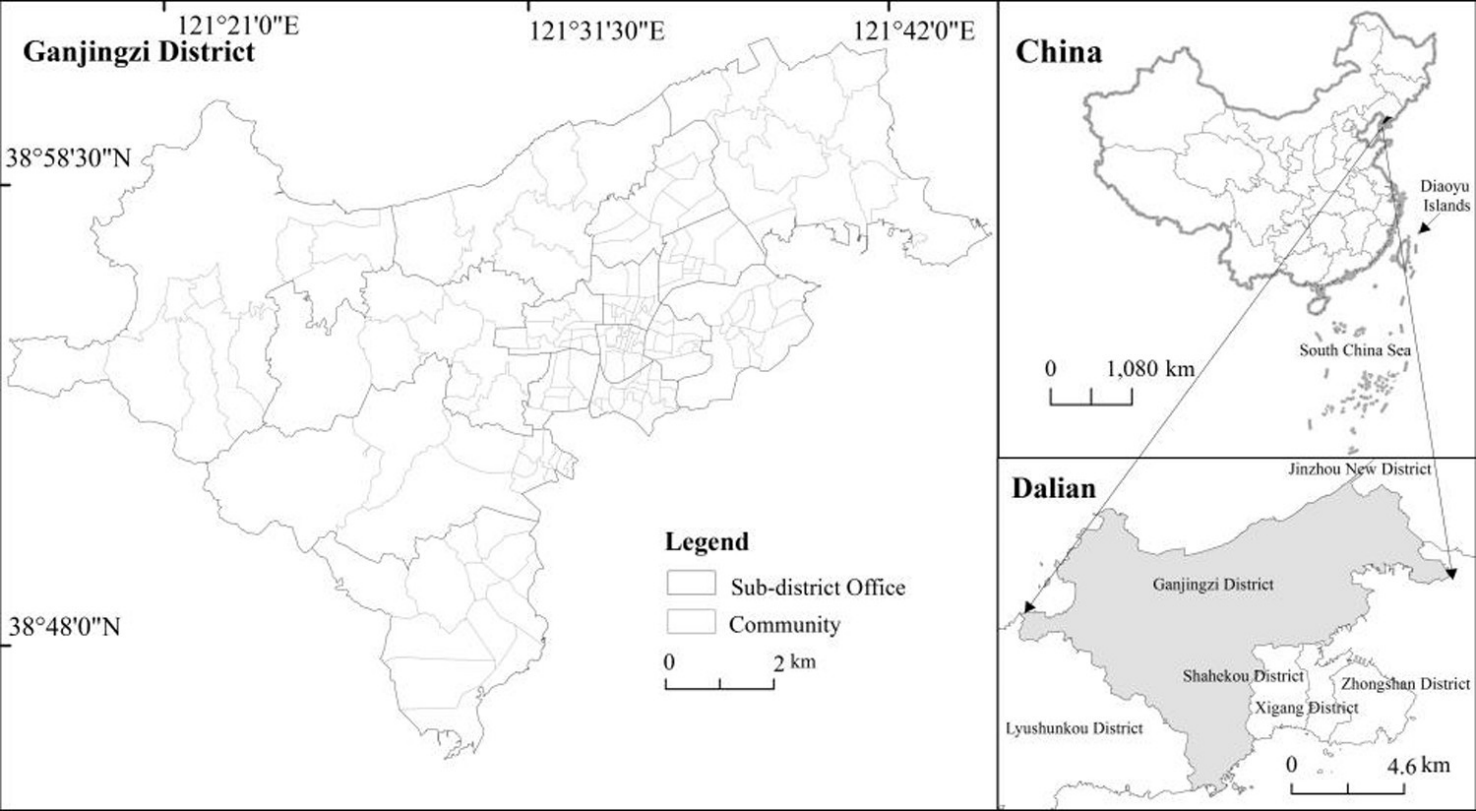
Location of study area: Ganjingzi District, in the city of Dalian in Liaoning Province, China. This map is drawn by the authors. This map was using ArcGIS ®software by Esri,©National platform for Common Geospatial Information Services, Creative Commons Attribution 4.0(CC BY 4.0).

Ganjingzi district (approximately 38°47′N–39°07′N, 121°16′E–121°45′E) is located in the downtown area of Dalian, with a horseshoe shape, bordering Jinpu, a new district in the northeast and Shahekou district in the south (Fig 1). As a neighbor, the southwest is connected to the Lushunkou district, covering an area of 502 km^2^, governing 14 streets, and 165 communities as the urban-rural integration area and urban expansion area of Dalian. The special geographical location makes the ecological service value of urban green space system of the Ganjingzi district change, gathers and is representative of the process of urbanization, and the research has important practical significance.

### Green space classification and classification standards

Based on land use data in 2000, SPOT5 data in 2006, and remote sensing image data from the second survey in 2012 and 2018, and based on ENVI and ArcGIS software, four time series land use data were obtained (Table 1). Combining the standards of "Land Use Classification" (GB/T2101-2007) and the characteristics of the study area, and based on the research results related to the impact of land use change on the value of ecosystem services, the soil and green land in Ganjingzi district is divided into four categories, that is, cultivated land, forest land, grassland, and swamp land (Table 2).

**Table 1:** Data sources and descriptions.

Data type	Data	Data source
Remote-sensing image data	Land use data 2000–2018(vector data)	Dalian Municipal Bureau of Land Resources and Housing
	2006 Dalian SPOT5 remote sensing data (resolution 2.5 m), multispectral images (resolution 10 m)	Dalian Municipal Bureau of Land Resources and Housing
	2012 Dalian resource 02C remote sensing data (resolution 2.5 m), multispectral images (resolution 10 m)	National Marine Environmental Monitoring Center
	2018 Dalian resource 02C remote sensing data (resolution 2.5 m), multispectral images (resolution 10 m)	National Marine Environmental Monitoring Center
Administrative data	Data of state, province, city, county (district), and village (town, sub-district)	Dalian Planning Bureau
Agricultural production data	National grain production data, Ganjingzi grain production data over the same period (2000–2018)	Dalian Statistical Yearbook (2000–2018), Ganjingzi Statistical Yearbook (2000–2018)

**Table 2:** Greenspace classification and content.

Classification	Content and scope
Forest	Refers to the land where trees, bamboos and shrubs grow, and the land where mangroves grow along the coast. Including relics, excluding land for greening forests within residential areas, forests within the scope of land acquisition by railways and highways, and embankment forests for rivers and ditches.
Arable land	Refers to the land where crops are grown, including cultivated land, newly developed, reclaimed, and reorganized land, fallow land (including rotation land and cropping land); mainly planted crops (including vegetables), with scattered fruit trees (mulberries or other trees); on average, one season of cultivated beaches and tidal flats can be harvested every year. Cultivated land includes fixed ditches, canals, roads, and ridges with a width of less than 1.0 m in the south and a width of less than 2.0 m in the north; cultivated land for temporary planting of medicinal materials, turf, flowers, seedlings, etc., and other cultivated land temporarily changed.
Grassland	Refers to the land where herbaceous plants grow.
Wetland	Refers to the land where there is frequent accumulation of water or waterlogging, and generally growing marsh and wet plants.

### Model method

#### Analysis of green landscape composition

**Fig 2:**
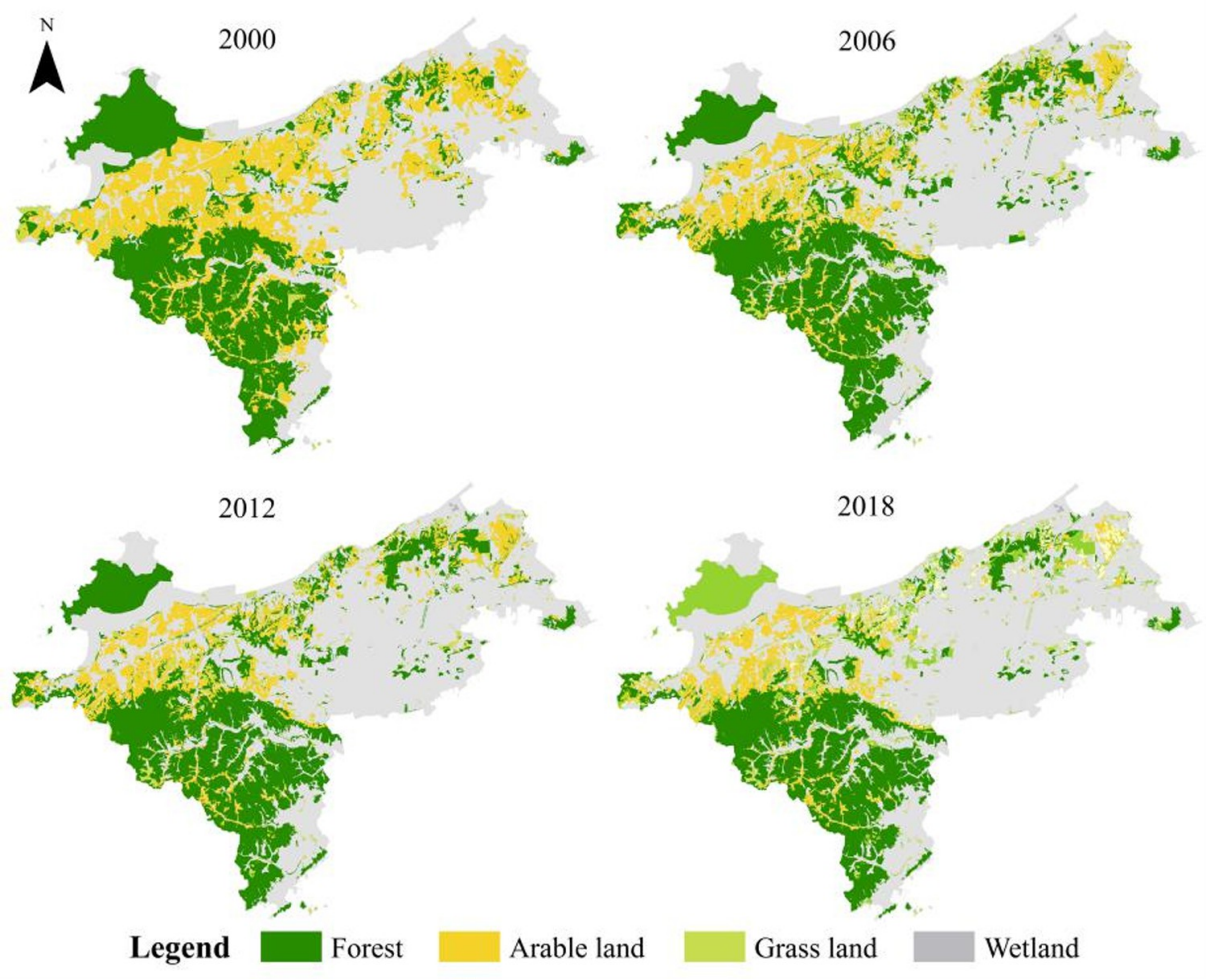
Variation of green space landscape in Ganjingzi District (2000–2018). This map is drawn by the authors. This map was using ArcGIS ®software by Esri,©National platform for Common Geospatial Information Services, Creative Commons Attribution 4.0(CC BY 4.0).

**Fig 3:**
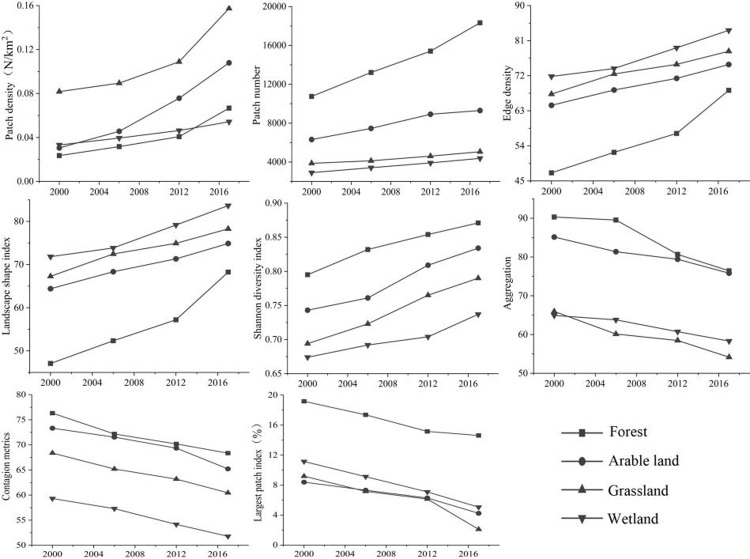
Variation of green space landscape indices in Ganjingzi District (2000–2018).

With the support of software like ENVI, ArcGIS, Fragstats4.3, and through remote sensing interpretation, manual classification and field investigation, the research area green space system was divided into four categories: cultivated land, woodland, grassland, and swamp, and finally obtained 2000, 2006, 2012, 2018 green space type map (Fig 2). In this study, various types of green space patch density (PD), patch number (NP), edge density (ED), landscape shape index (LSI), Shannon diversity index (SHDI), aggregation degree (AI), contagion metrics degree (CONTA), and the largest patch area index (LPI), were used to reflect the evolution characteristics of the green space fragmentation in the study area, and quantitatively analyze the fragmentation evolution of the green space landscape pattern in Ganjingzi district (Fig 3).

*Evaluation method of green space ecosystem service value*. This study is based on the "Natural Cost Method" proposed by Costanz [37], combined with the "China Ecosystem Service Value Equivalent Factor Table" by Xie [28, 43], and draws on the results of previous studies to investigate the area and the national grain per unit area. The ratio of output and the correction coefficient are determined, to evaluate the service value per unit area of the green space system in Ganjingzi district of Dalian (Table 3).

**Table 3:** Coefficient of ecology service value of green space (unit:yuan/km2•yr).

	Forest	Arable land	Grass land	Wet land
Gas regulation	203,423	22,121	45,764	51,740
Climate regulation	198,914	34,314	94,631	55,183
Water conservation	203,123	26,543	153,044	88,642
Soil formation and conservation	285,054	65,035	143,412	33,956
Waste disposal	65,922	73,046	101,432	34,945
Biodiversity and conservation	248,461	30,119	73,645	84,313
Food production	8,853	45,092	34,217	5,736
Raw material supply	140,064	3,043	9,367	5,232
Recreation and culture	83,260	413	60,143	1,311

The evaluation formula of ecosystem service function value is as follows:

$$x=\frac{a}{A},$$ (1)

where *x* is the correction coefficient, *a* is the annual grain output per unit of Ganjingzi district (kg/km^2^), and *A* is the national grain output per unit area kg/km^2^ during the same period.

$$\begin{array}{l} ESV_{k}=A_{k}\times VC_{k}\\ ESV_{t}=\sum_{k}A_{k}\times VC_{k}\\ ESV_{f}=\sum_{k}A_{k}\times VC_{kf}, \end{array}$$ (2)

where *ESV*_*k*_ is the ecological service value of green space type *k*, *ESV*_*f*_ is the ecological service value of ecological function *f*, *ESV*_*t*_ is the total ecological service value of green space system, *A*_*k*_ is the area of green space type *k* (km^2^), and *VC*_*k*_ and *VC*_*kf*_ are the ecology of green space system service value coefficient and ecological service function value coefficient, respectively.

#### Dynamic degree of service value change of green space ecosystem

The ecological service value dynamic describes the change in speed of the ecological service value of a certain type of green space in a particular period [44], the formula is as follows:

$$ESV_{k}=\frac{ESV_{b}‐ESV_{a}}{ESV_{a}}\times \frac{1}{T},$$ (3)

where *ESV*_*a*_ is the ecological service value of a certain type of green space at the beginning of the study, *ESV*_*b*_ is the ecological service value of the green area at the end of the study, and *T* is the study period. If *ESV*_*k*_ > 0, the value of ecological services will increase; if *ESV*_*k*_ < 0, the value of ecological services will decrease; and if *ESV*_*k*_ = 0, the value of ecological services will remain unchanged.

With reference to relevant research results and actual conditions, the dynamics of green space ecosystem service value changes are divided into seven categories: significant reduction -0.35 ≤ *ESV*_*k*_ < -0.25, moderate reduction -0.25 ≤ *ESV*_*k*_ < -0.15, mild reduction -0.15 ≤ *ESV*_*k*_ < 0, no change *ESV*_*k*_ = 0, mild increase 0 <*ESV*_*k*_ ≤ 0.15, moderate increase 0.15 < *ESV*_*k*_ ≤ 0.25, and significant increase 0.25 < *ESV*_*k*_ ≤ 0.35.

#### Impact of fragmentation of green space on ecological service value

Considering the green space landscape pattern index as an independent variable, the value of each ecological service function and the total value of the green space as the dependent variables By using SPSS software to analyze the correlation of the research variables, and the effect of the fragmentation evolution of each type of green space on the value of the ecological service function is obtained [45]. The influence results are presented (Table 4).

**Table 4:** Correlation of greenspace pattern change and ecosystem service value structure.

	Forest								Arable land							
	PD	NP	ED	LSI	SHDI	AI	CONTA	LPI	PD	NP	ED	LSI	SHDI	AI	CONTA	LPI
Regulation function	−0.843	−0.866	−0.893	−0.813	−0.792	0.833*	0.704**	0.781**	−0.782	−0.713	−0.673	−0.691	−0.742	0.774**	0.761**	0.753*
Support function	−0.599	−0.632	−0.561	−0.592	−0.614	0.657	0.736	0.516	−0.764	−0.739*	−0.758**	−0.752*	−0.784*	0.764**	0.720**	0.675
Supply function	−0.422	−0.547	−0.605	−0.567	−0.506	0.395	0.371	0.401	−0.489	−0.553	−0.668**	−0.675**	−0.652**	0.556	0.603	0.518
Recreation function	−0.248	−0.271	−0.184	−0.193	−0.188	0.172	0.195	0.263	−0.275	−0.286	−0.170	−0.243	−0.178	0.231	0.194	0.296
	Grass land								Wetland							
	PD	NP	ED	LSI	SHDI	AI	CONTA	LPI	PD	NP	ED	LSI	SHDI	AI	CONTA	LPI
Regulation function	−0.831*	−0.764	−0.815*	−0.822**	−0.821*	0.782	0.764	0.812	−0.764*	−0.831**	−0.846	−0.672	−0.703	0.645**	0.703**	0.751
Support function	−0.750	−0.761	−0.640	−0.638	−0.703*	0.682*	0.693**	0.734*	−0.652	−0.674**	−0.733*	−0.594**	−0.564*	0.604**	0.567**	0.693
Supply function	−0.307	−0.419	−0.294	−0.284	−0.295	0.285	0.286	0.327	−0.641	−0.594	−0.462	−0.532	−0.516	0.533	0.652	0.692
Recreation function	−0.302	−0.236	−0.251	−0.261	−0.315	0.196	0.185	0.215	−0.217	−0.432	−0.526	−0.495	−0.380	0.175	0.184	0.275

**p≤0.01

*p≤0.05.

## Results

### Analysis of the change of the green landscape composition

The land use structure of Ganjingzi district changed during the period from 2000 to 2018. With the gradual expansion of urban construction land, the area of urban green space was gradually reduced (Fig 2). The total green area reduced from 359.57 to 213.46 km^2^. The woodland, arable land, and grassland are concentrated in the west, southwest, north and northeast of Ganjingzi district. This part of the area is mainly hilly and mountainous terrain, which is affected by topography. The expansion of urban construction land in concentrated green areas is relatively slow. The green areas such as woodland, arable land, and grassland are concentrated and contiguous. The area of the most extensive and concentrated forest land has been reduced from 212.72 to 155.78 km^2^. During the process of urbanization in the central part of Ganjingzi, the urban construction area increased, and the urban population expanded, gradually occupying and reducing the area of urban green space and causing the urban area to gradually shrink and fragment. The scattered cultivated land, grassland and other types of green land in the central part of China decreased rapidly from 2006 to 2018. The area of arable land was reduced from 32.3 to 16.46 km^2^, and the area of grassland was reduced from 29.72 to 13.06 km^2^.

Analysis of changes in the green landscape index of Ganjingzi district from 2000 to 2018 (Fig 3). From 2000 to 2018, the green landscape of Ganjingzi district has undergone significant changes. Urban green PD, patch number, edge density, landscape shape index, and SHDI have increased every year; aggregation, spread, and maximum patch index have slowly decreased. Because the urbanization process is accelerating, the urban population and urban construction are gradually expanding to the urban fringe area, and the urban construction area is gradually increasing. Therefore, the total area of the green landscape in Ganjingzi district is not only significantly reduced, but also gradually fragmented. From 2012 to 2018, with the deepening of the concept of sustainable development and the implementation of ecological city construction policies and implementation, attention has been paid to the ecological sustainable construction of urban fringe areas. While developing urban construction in urban fringe areas, attention has been paid to ecological environmental protection and protection of the original green space system, to increase the area of artificial green space, alleviate the gradual fragmentation of the green space system, restore the ecological service function of the green space, and increase the value of the ecological service of the green space.

### Analysis on the spatio-temporal evolution of ecological service value of green space

#### Analysis on the evolution of ecological function value of green space type

**Fig 4:**
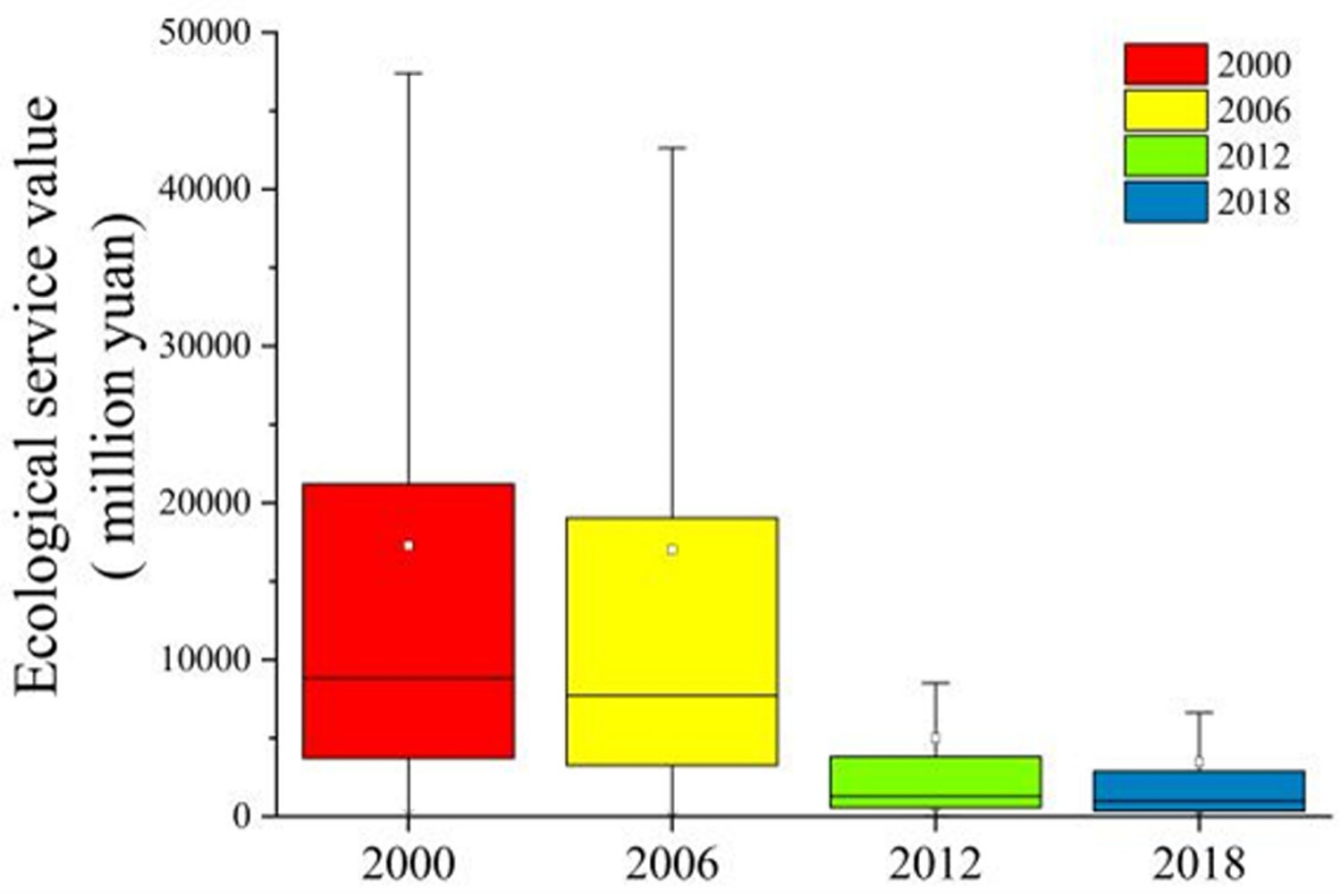
Variation of green space landscape ecosystem service value in Ganjingzi District (2000–2018).

With the continuous reduction of the green landscape area in Ganjingzi district, the landscape is gradually fragmented, and the value of green space ecological services is gradually decreasing (Fig 4). In 2000, the total area of green land was the largest, with the highest green land ecological service value of 396.27 million yuan, and the average ecological service value of 50.12 million yuan. The ecological service value of most green land patches was above the average ecological service value, and the ecological service value of green land was higher. The value of ecological services increased during 2006 to 2012, with the highest value increasing from 174.62 to 213.09 million yuan. However, the value of ecological services decreased rapidly during 2012 to 2018, with the highest value being reduced from 213.09 to 120.04 million yuan. The average ecological service value during 2006 to 2018 was low, the highest value gradually decreased, and the green space ecological service value was generally low.

**Fig 5:**
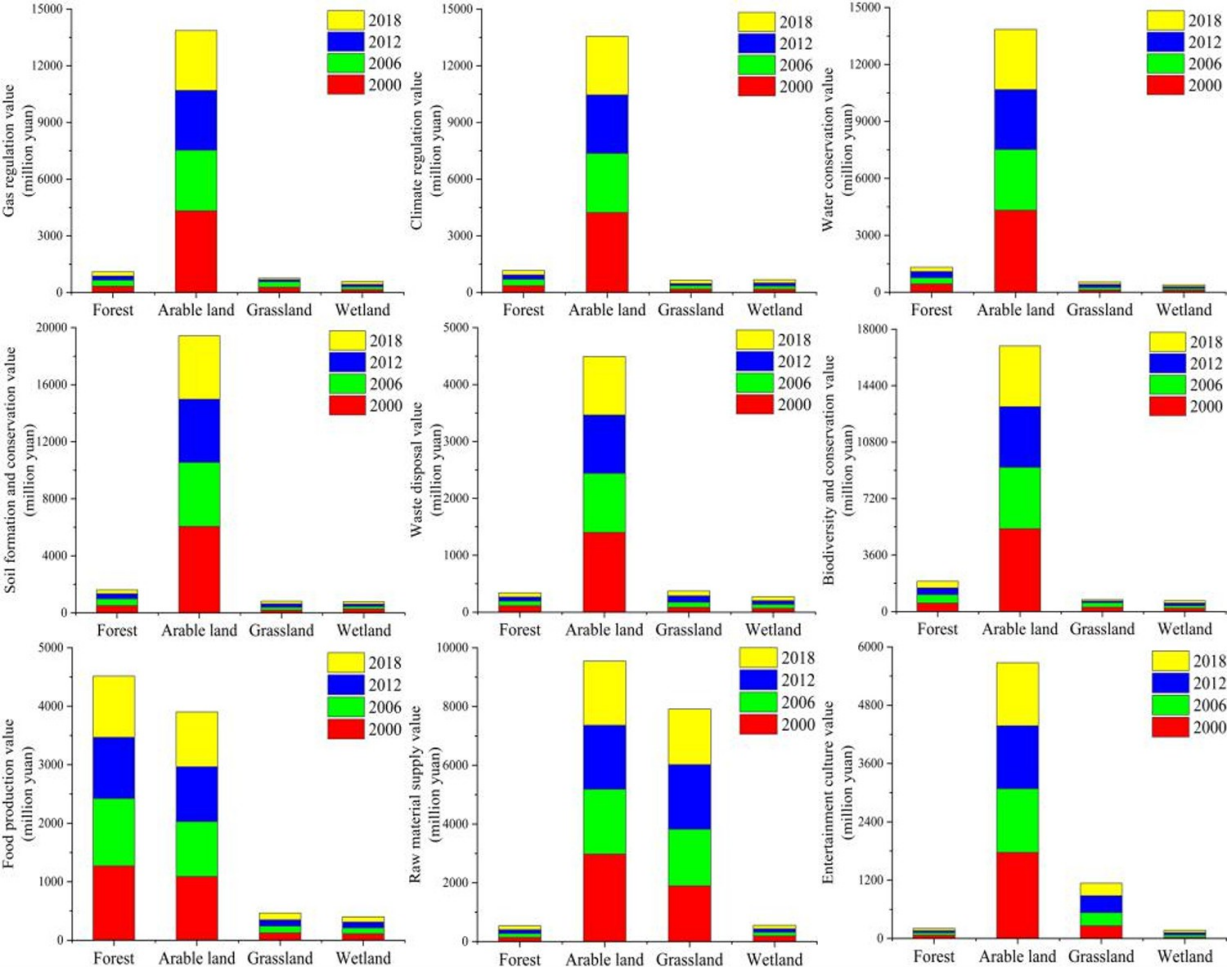
Variation of green space ecosystem service and function values in Ganjingzi District (2000–2018).

Ganjingzi district has a large area of woodland, that has higher gas and climate regulation value than other types of green land. From 2000 to 2018, the gas and climate regulation functions of forest land had the highest value and greatest impact among all types of green land (Fig 5). The value of the gas regulation function of woodland has dropped from 2.4161 to 3.3319 million yuan; the value of climate regulation has dropped from 2.3625 to 0.77 million yuan, which is a significant decrease. The climate regulation function value of other green space types is generally low, and the changes are relatively small.

Ganjingzi district is Dalian’s urban fringe and urban-rural integration area. Agriculture occupies a large proportion of the industrial structure and has large arable lands. Compared with other types of green space, this region has more ecological support function value. From 2000 to 2018, the value of water conservation function of cultivated land decreased from 0.34 to 0.25 million yuan; the value of soil formation and protection decreased from 0.81 to 0.39 million yuan; and the value of waste disposal decreased from 0.86 to 0.35 million yuan. The various ecological service functions of forest land have the highest value and the greatest impact among all types of green land. The value of water conservation function of forest land decreased from 2.41 to 2.39 million yuan; the value of biodiversity and protection decreased from 2.95 to 0.97 million yuan; which was a significant decrease. The ecological purification and supporting functions of grassland and swamps were generally of low value, and the changes were relatively small.

Cultivated land has more ecological function value for product supply than other types of green land. The food production value of arable land gradually decreased from 0.78 to 0.49 million yuan during the period from 2000 to 2018. The food production value and raw material production value of forest land declined rapidly during 2000 to 2006 and was relatively stable during 2006 to 2018.

Ganjingzi district has a good ecological environment. Ecological entertainment parks such as Hongqi Valley Golf Club and Dalian Jinlong Temple Forest Park are in the southeast and northeast of Ganjingzi district. The woodlands and swamps in Ganjingzi district have relatively high entertainment and cultural value.

#### Analysis on the spatial evolution of green space ecological service value

**Fig 6:**
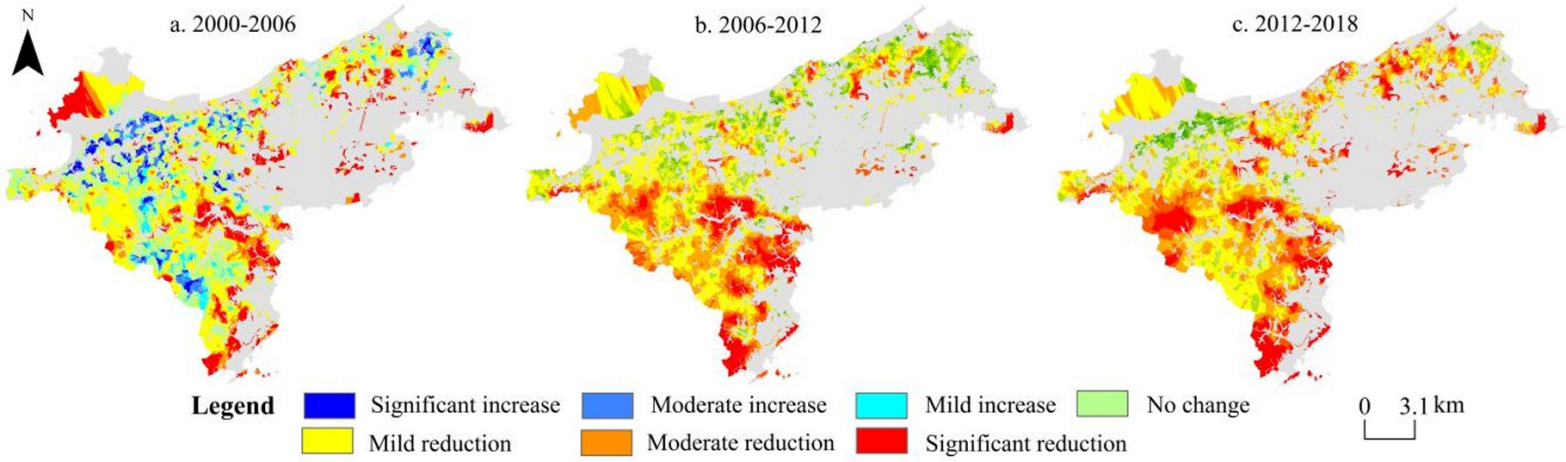
Spatial distribution of change of green ecological service value (2000–2018). This map is drawn by the authors. This map was using ArcGIS ®software by Esri,©National platform for Common Geospatial Information Services, Creative Commons Attribution 4.0(CC BY 4.0).

From 2000 to 2018, the ecological service value of green space in Ganjingzi district showed a slight increase and then a significant decrease (Fig 6). With the continuous advancement of urbanization, the ecological service value of various types of green landscapes shows different characteristics of change. From 2000 to 2006, the suburban agriculture in Ganjingzi district developed rapidly. The area of arable land increased, the cultivation method was intensively developed, and the dynamics of changes in ecological service value increased. The highest dynamic was 0.294, and the area of grassland and swamps decreased. The dynamics of changes in the value of ecological services decreased, with the lowest dynamics of -0.349. The urbanization process progressed rapidly during 2006 to 2018, and the urban population increased sharply. As an urban expansion area of Dalian, Ganjingzi district has gradually transformed its industry and land use. The structure is rapidly changing, the urban construction land is increasing and eroding the green landscape, due to which it is getting severely fragmented, and the dynamics of changes in the ecological service value of various types of these lands are showing a negative growth state.

### Analysis on the impact of fragmentation of green space on ecological service value

In Ganjingzi district during 2000 to 2018, the urban construction land gradually expanded and eroded the urban green space, resulting in the transformation of the urban green space landscape from completeness and high connectivity to boundary fragmentation and fragmentation. SPSS was used to analyze the correlation coefficients between changes in the landscape pattern of green spaces and changes in the value of various ecological functions of green spaces (Table 4). The value of various ecological service functions of green space is negatively correlated with PD, patch number (NP), ED, LSI, and SHDI of the green landscape. There is a positive correlation with the degree of aggregation (AI), degree of contagion metrics (CONTA), and maximum plaque area index (LPI).

Through correlation analysis, during 2000 to 2018, the ecological regulation kinetic energy and supporting functions of the green land in Ganjingzi district have a relatively high correlation coefficient to the evolution of the green scape, while the ecological supply function and entertainment function have a low correlation coefficient to the evolution of the green scape.

The adjustment function and support function value of the green space system are highly correlated with the evolution of the landscape pattern, and the highest adjustment function index is 0.866. As the area of the green space landscape shrinks and gradually fragments, the ecological functions of the green space system such as gas regulation, climate regulation, and water conservation have gradually weakened. The production function and entertainment cultural function value of the green space system has a small correlation index to the change of the green space landscape index. The production and development models of each green space type have changed and improved during 2000 and 2018, and the green space is gradually shrinking, and the landscape is gradually fragmented. Under the circumstances of globalization, production functions, entertainment and cultural functions have not been greatly affected, and the value of ecological services generated by functions such as food and fruit planting and green space landscape entertainment has increased.

In general, the ecological service value of woodland and marshland has a high correlation with the change of green space landscape pattern, and the correlation index of regulation function is the highest. The correlation index of regulation function value of woodland and patch quantity index evolution is -0.866. The correlation index between the adjustment function value of the marshland and the evolution of the plate number index is -0.892. The correlation index between the ecological service value of cultivated land and grassland and the evolution of the green space landscape pattern is relatively small, and the correlation index between the production function value of cultivated land and the evolution of patch area index is 0.402.

## Discussion

### Value equivalent adjustment

Based on the traditional value equivalent method, this paper evaluates the ecological service value of urban fringe areas and analyzes the impact of the fragmentation of the green landscape on the ecological service value [46, 47]. Ecological service value evaluation methods are diverse, research space scales are different, and domestic and foreign scholars have different classification standards for ecological service functions, which make the ecological service value equivalent factor not applicable to all research scales and research areas [48]. For specific small-scale research areas, it is necessary to adjust the national average ecological service value equivalent factors of Xie and others [49]. Therefore, the ratio of grain output per unit area of the study area in this article to the country is evaluated to determine the correction coefficient, and then to determine the green space in Ganjingzi district service value per unit area of the system.

### Sensitivity analysis of green space ESV

To clarify the degree of dependence of the ecological service value on the change of the value coefficient and test the impact of the adjusted value equivalent factor on the evaluation of the ecological service value [12, 23], this paper uses the concept of elasticity coefficient to calculate the sensitivity index of the ecological service value to the value coefficient and further determine the value of the accuracy of the coefficient. The coefficient of sensitivity formula is as follows:

CS=|(ESVj−ESVi)ESVi(VCjk−VCik)VCik|, (4)

where, *ESV*, *VC* and *k* have the same meaning as formula (2), and *i* and *j* represent before and after adjustment. If *CS* > 1, it means that *ESV* is sensitive and flexible to *VC*; if *CS* < 1, the opposite is true. The smaller the *CS* value, the less decisive *VC* is for *ESV*. The smaller the *CS* value, the smaller the impact of the value coefficient of the green space type on the ecological service value of the green space system. The closer the value coefficient selected to the service value of the green space system in the study, the better the fit.

**Fig 7:**
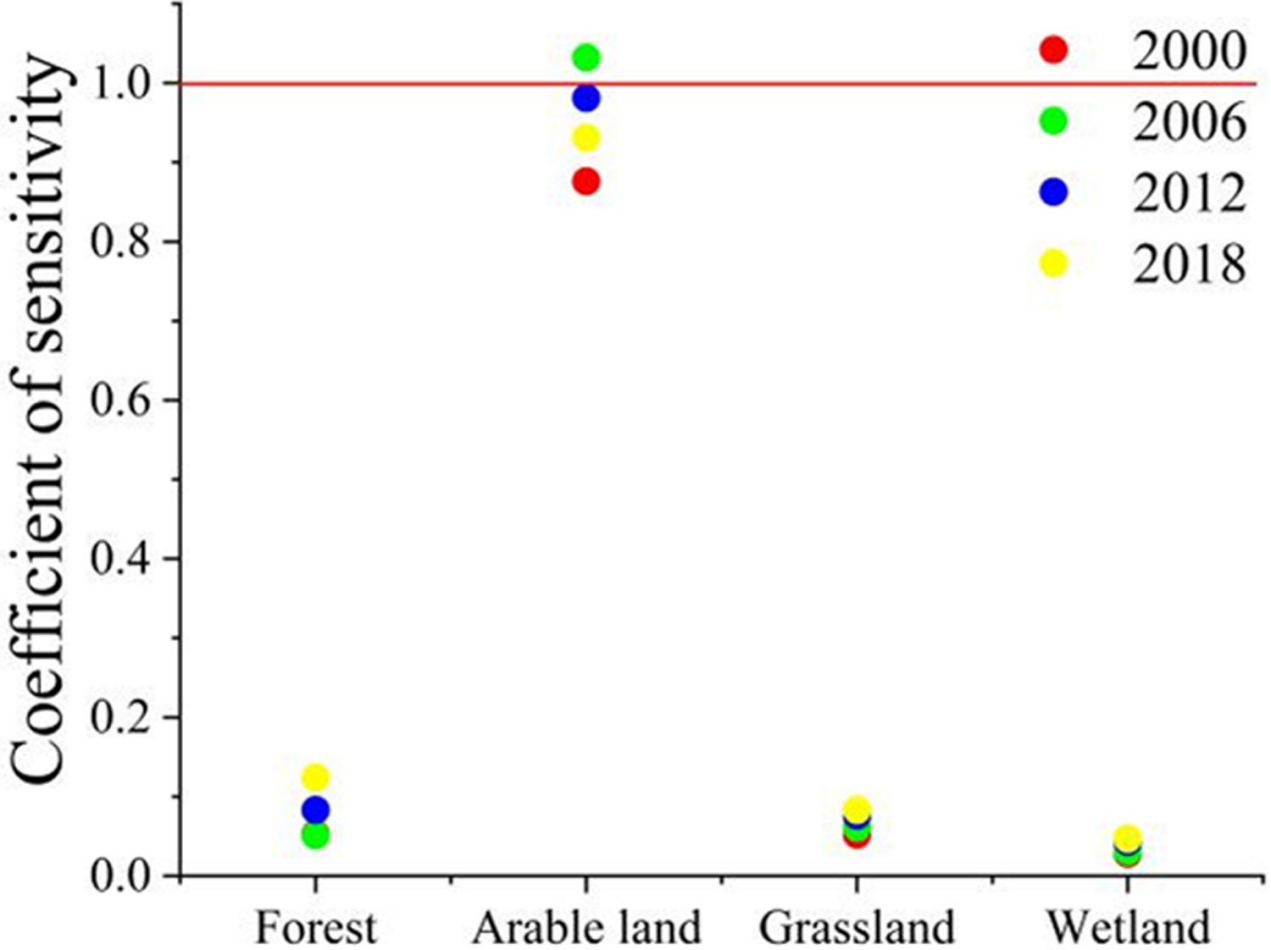
Distribution of coefficient of sensitivity of green space ecosystem service values (2000–2018).

Through the sensitivity analysis, the distribution chart of the sensitivity coefficient of green land ecological service value (Table 5 and Fig 7) is obtained. The sensitivity index of forest land in 2018 was 1.031. The grass has the lowest sensitivity. Except for forest land, the sensitivity coefficients of other green land types are all less than 1, and the value coefficients have relatively little impact on the value of ecological services, and the estimated value of ecological services lacks flexibility. Therefore, the value coefficient selected by the research is relatively reasonable and appropriate for the value of green space landscape ecological services.

**Table 5:** Coefficient of sensitivity of greenspace ESV in Ganjingzi District (2000–2018).

	Forest	Arable land	Grass land	Wetland
2000	0.876	0.053	0.052	0.028
2006	1.032	0.051	0.061	0.031
2012	0.981	0.083	0.076	0.043
2018	0.931	0.124	0.083	0.047

### Urban fringes ecological recovery advice

Through the study, in the process of urban development, Ganjingzi District has gradually positioned the urban fringes and urban development zone in 2000–2017. During the development, the expansion of urban construction land will continue to extrusion urban green space system, resulting in crushing of urban green space, making the ecological service value of urban green space [34, 49]. Dalian City, urban ecological restoration construction should gradually restore urban ecological green space, repair the original forest grass, and increase the area of artificial green space, thereby enhancing urban green space adjustment function and support function. Strengthening the green space ecological environment of Ganjingzi District, and change the cultivation model to improve the value of the production of green space systems on the basis of ensuring the original cultivar area. In the process of urban development, the green space is unavoidable. In order to promote the sustainable development of urban ecological environment, urban green space protection should be strengthened, enhance urban green space utilization efficiency, and improve urban ecological service value.

## Conclusion

As the carrying space for urban spatial and population expansion, urban fringe areas are faced with urban problems such as the transformation of landscape structure, the reduction of green space, and the weakening of green landscape ecological service functions in the process of urbanization. This study uses the urban fringe area of Dalian as the research area, based on 2000–2018. Ganjingzi district land use data and remote sensing image data, analyzes various green space landscape changes, evaluates the green space ecological service value and its change characteristics, and the structure shows:

1) From the analysis of the green landscape composition, from 2000 to 2018, with the advancement of urbanization, the area of construction land continued to increase, the area of green land continued to shrink, and the landscape gradually fragmented. The total green area has been reduced from 359.57 to 213.46 km^2^. Among them, the area of woodland has decreased the most, from 212.72 to 105.78 km^2^, and other types of green space are gradually decreasing.

2) From the analysis of the ecological service function value of green land and the temporal and spatial evolution characteristics of the change of ecological service value, the value of green land ecological service gradually decreased from 397.42 to 124.93 million yuan during the period from 2000 to 2018. The value of ecological service functions of various types of green land is decreasing every year. Among various types of green land, the value of ecological service functions of forest land and cultivated land is higher. From the perspective of spatial change characteristics, the dynamics of ecological service value changes in the western and northern regions with a relatively concentrated green space are higher than those in the eastern regions. The value of ecological services recovered during 2000–2006, and the value of ecological services continued to decrease during 2006 to 2018.

3) From the analysis of the correlation between the value of ecological service and the change of green space landscape, the value of the ecological service function of the green space system affects PD, patch number (NP), ED, LSI, and variety Sex index (SHDI) and is negatively correlated; it is positively correlated with aggregation degree (AI), contagion (CONTA), and maximum plaque area index (LPI). Regulating and supporting functions have a higher correlation index with green landscape fragmentation, and the correlation coefficient between the regulation function of woodland and the evolution of patch number index is the highest (-0.874); the correlation index between production function, entertainment and cultural functions, and green landscape fragmentation is higher The correlation coefficient of the entertainment and cultural service function of the marshland on the evolution of the PD index is the lowest (-0.214).
